# Risk factors associated with gestational transient thyrotoxicosis and their effects on the pregnancy course

**DOI:** 10.20945/2359-4292-2024-0129

**Published:** 2025-04-03

**Authors:** Bagdagul Yuksel Guler, Ibrahim Demirci, Cem Haymana, Alper Sonmez, Ahmet Faruk Yagci

**Affiliations:** 1 Department of Endocrinology and Metabolism, Sincan Education and Research Hospital, Ankara, Türkiye; 2 Department of Endocrinology and Metabolism, Gulhane School of Medicine, University of Health Sciences, Etlik, Ankara, Türkiye; 3 Department of Cardiology, Gulhane School of Medicine, University of Health Sciences, Etlik, Ankara, Türkiye; 4 Department of Endocrinology and Metabolism, Ankara Guven Hospital, Türkiye

**Keywords:** Gestational transient thyrotoxicosis, Abortion, Thyroid function tests, Pregnancy

## Abstract

**Objective:**

To investigate the clinical characteristics of patients with gestational transient thyrotoxicosis and its possible impacts on pregnancy.

**Methods:**

This retrospective study included pregnant women with gestational transient thyrotoxicosis who were admitted to our endocrinology outpatient clinic from June 2020 to March 2023. Patients with other causes of thyrotoxicosis, such as Graves’ disease, toxic nodular goiter, and subacute thyroiditis, were excluded.

**Results:**

The study included 50 pregnant women who met the inclusion criteria and whose data could be accessed. Two pregnant women were diagnosed with gestational diabetes, and two pregnancies resulted in abortion. We observed that thyroid-stimulating hormone levels normalized to euthyroid values at a mean gestational age of 18.3 ± 3.7 weeks. The mean gestational age at birth was 38 ± 1.8 weeks. The frequency of preterm labor, defined as delivery before 37 weeks, was 10% (n = 5). Sinus rhythm was observed in 87% of the electrocardiograms obtained during thyrotoxicosis, while sinus tachycardia was detected in four and sinus arrhythmia in two cases. Thyroid nodules were observed in 23 (47.9%) of 48 cases in which ultrasonography was performed during thyrotoxicosis. Discussion: This retrospective study, including 50 pregnant women with gestational transient thyrotoxicosis, found no increase in the rate of serious obstetric complications such as eclampsia/preeclampsia, gestational diabetes, preterm labor, or abortion. Notably, in a detailed examination of electrocardiograms, which has not been done in previous studies, we did not detect any serious, life-threatening arrhythmias, although tachycardia was observed.

## INTRODUCTION

Gestational transient thyrotoxicosis (GTT) is a form of hyperthyroidism that develops due to the thyroid-stimulating effects of human chorionic gonadotropin (hCG) secreted from the placenta during the early weeks of pregnancy (^1^). First described by Kimura et al. in 1993, GTT occurs in the first trimester of pregnancy and usually resolves spontaneously in the second trimester - often early or by the middle of this period (^2^). While the frequency of true hyperthyroidism in pregnancy is reported to be 0.05%, the GTT frequency is estimated to vary between 2 and 11% (^3,4^). As mentioned, the stimulation of the thyroid gland by placental hCG is a crucial step in GTT; however, various other factors have been suggested to contribute to GTT development, including dysregulation of hCG production, hypersensitivity of thyroid-stimulating hormone (TSH) receptors to hCG, and prolonged sensitivity of the thyroid gland to thyroid hormone stimulation (^5^).

The majority of pregnant women who develop GTT are asymptomatic; however, some patients may present with symptoms and findings such as tachycardia, increased sweating, anxiety, irritability, and weight loss (^6^). The literature on this topic appears to largely agree that GTT has no adverse effects on maternal or fetal outcomes, although some publications report contrary results (^7^). Some case reports have revealed severe morbidities attributed to GTT, including acute myocardial infarction and fatal arrhythmia (^8^), as well as acute liver injury requiring therapeutic plasma exchange (^9^). There are also publications reporting that GTT increases the likelihood of gestational diabetes mellitus (GDM) (^10^).

The primary aim of this study was to investigate the clinical characteristics of patients with GTT and its possible impacts on pregnancy. Secondarily, we aimed to identify the cardiac outcomes associated with GTT and its effects on the frequency of GDM.

## METHODS

This was a retrospective study including pregnant women who had been admitted to our endocrinology outpatient clinic with a diagnosis of GTT from June 2020 until March 2023. Patients with other causes of thyrotoxicosis, such as Graves’ disease, toxic nodular goiter, or subacute thyroiditis, were excluded. Age, comorbidities, and obstetric data comprising the number of pregnancies, living children, abortions, and stillbirths (if any), along with history of nausea and/or vomiting requiring medication in the current and previous pregnancies were recorded. The height and body weight of all pregnant women were measured and recorded. Body mass index was calculated by dividing body weight (kg) by height squared (m^2^; kg/m^2^). Additionally, patient files and digital records were assessed to determine whether an oral glucose tolerance test was performed to screen for GDM during pregnancy, and, if so, the results were evaluated and included in the analyses. After the patient gave birth, the gestational week at birth and any adverse pregnancy outcomes, such as preterm birth (delivery before 37 weeks), low birth weight, or stillbirth, were also recorded.

Blood pressure and heart rate measurements are routinely performed on every pregnant woman admitted to our outpatient clinic with a suspicion of thyrotoxicosis, including GTT. Blood pressure measurements were performed using an Omron M2 Basic (HEM-7121J-E; Omron Healthcare Co., Ltd., Kyoto, Japan) device, an upper-arm digital blood pressure monitor used in our outpatient clinic. The electrocardiograms (ECGs) of patients were obtained from patient files or digital records, and the results of all patients were interpreted by the same cardiologist. In each ECG, the cardiologist assessed the rhythm (sinus or non-sinus), heart rate (beats/minute), supraventricular and ventricular extrasystoles, axis, ventricular hypertrophy, atrial enlargement, P wave, PR and QT intervals, corrected QT interval (QTc), ischemic changes, low QRS voltage, and loss of R-wave progression.

Each pregnant woman with a preliminary diagnosis of GTT underwent thyroid ultrasonography performed by endocrinologists in our outpatient clinic using an Esaote ultrasound device (Esaote S.p.A., Genova, Italy). Thyroid volume and the presence of nodules were assessed during the ultrasonographic evaluation. Thyroid volume was calculated using the formula volume = length × width × depth × correction factor. The correction factor was 0.479, which is used by many organizations, including the World Health Organization (WHO) (^11^).

The levels of free triiodothyronine (fT3), free thyroxine (fT4), TSH, TSH receptor antibody (TRAb), antithyroglobulin antibody (anti-Tg), antithyroid peroxidase antibody (anti- TPO), beta hCG, alanine aminotransferase (ALT), and aspartate transaminase (AST) were recorded. The fT3, fT4, TSH, anti-TPO, and anti-Tg measurements were performed using a Cobas e 801 analyzer (Roche Diagnostics, Mannheim, Germany) with an electrochemiluminescence immunoassay. Levels of ALT and AST were measured using an enzymatic method with a Cobas c 701 analyzer (Roche Diagnostics). The presence of TRAb was measured using a second-generation assay employing the electrochemiluminescence immunoassay methodology (Cobas Pro, Roche Diagnostics), with results < 1.0 IU/L considered negative. Levels of TSH were evaluated based on reference values recommended in obstetric guidelines. The lower reference limit for TSH was 0.1 mIU/L for the first trimester, 0.2 mIU/L for the second trimester, and 0.3 mIU/L for the third trimester.

The present study was approved by the Ethics Committee of Gulhane Medical School (approval date: 31 August 2023, approval number: 2023/185). Statistical analyses were performed using Statistical Package for the Social Sciences (SPSS) software (version 20.0; IBM Corp, Armonk, NY, USA). Descriptive statistics were summarized as counts (n) and percentages (%) for categorical variables. Continuous variables with normal distribution are presented as mean and standard deviation values, while those without normal distribution are presented as median (interquartile range) values.

## RESULTS

The study included 50 pregnant women who met the inclusion criteria based on data availability. The mean age was 28.8 ± 6.2 years, the median gravidity was 2, and 28% of the pregnant women had a history of abortion. Additionally, 62% of participants reported having received treatment for nausea and vomiting. The mean blood pressure and heart rate values of the pregnant women were within reference ranges **(Table 1).**

**Table 1:** Demographic and obstetric data of pregnant women with gestational thyrotoxicosis

Variables	Pregnant women with gestational thyrotoxicosis (n = 50)
Patient age, years	28.8 ± 6.2
Gravidity	2 (^1-9^)
History of abortion	14 (28%)
History of thyrotoxicosis in previous pregnancies	6 (12%)
Body mass index, kg/m^2^	24.5 ± 4.0
18.5	4%
18.5-24.9	52%
25-29.9	18%
≥ 30	12%
SBP, mmHg	116 ± 10
DBP, mmHg	72 ± 7
Heart rate (beats per minute)	90 ± 12
Patients with thyroid nodules	23 (46%)

Results expressed as mean ± standard deviation, median (minimum-maximum) or n (%).

SBP: systolic blood pressure; DBP: diastolic blood pressure.

The mean gestational week at birth was 38 ± 1.8 weeks. Five (10%) patients experienced preterm labor, three of whom had multiple pregnancies. The data on the newborns are detailed in **Table 2.** Notably, there were two cases of abortion. The first occurred at 16 weeks of gestation in the 4th pregnancy of a 36-year-old woman who had a history of two abortions and one live birth. The TSH levels were suppressed at the time of abortion. The second occurred in the third pregnancy of a 32-year-old woman who had a history of two abortions. The TSH levels were within the reference range at the time of abortion. Finally, preeclampsia/eclampsia or pregnancy-induced hypertension were not detected in any pregnancy.

**Table 2:** Data on newborns of pregnant women with gestational thyrotoxicosis

Variables	Data on newborns (n = 51)
Gestational age at birth, weeks	38 ± 1.8
Preterm labor	5 (^10^)
Newborn birth weight, g	3,137 ± 495
Newborns with low birth weight	5 (9.8%)
Sex, female	25 (49%)
Cesarean delivery	20 (40%)
Multiple pregnancy	3 (6%)

Results expressed as mean ± standard deviation or n (%).

The mean gestational age at GTT diagnosis was 10.3 ± 2.5 weeks. While most pregnant women were subclinically thyrotoxic at the time of diagnosis, approximately 20% had elevated fT4 levels, which were between the upper limit of normal and 1.5 time higher. The TSH levels of 40 (80%) pregnant women reached the trimester-specific reference range for pregnancy at a mean of 18.3 ± 3.7 weeks. Among these patients, the latest recovery occurred by week 26. With respect to antibodies, anti-Tg and anti-TPO antibodies were detected in 8% and 12% of the women with GTT, respectively, while TRAb was negative in all patients. Finally, ALT and AST levels were within reference ranges in all pregnant women at the time of thyrotoxicosis **(Table 3)**.

**Table 3:** Laboratory results of the patients at first admission

Variables	Reference range	Patients with GTT (n=50)
fT_3_	2-4.4 pg/mL	4.3 ± 0.7
fT_4_	0.8-1.7 ng/dL	1.3 ± 0.4
TSH	0.4-4.2 µIU/mL	0.02 ± 0.02
Anti-TPO positivity	0-34 IU/mL	6 (12%)
Anti-Tg positivity	0-115 IU/mL	4 (8%)
TRAb positivity	< 1.0 IU/L	0
ALT	0-33 U/L	14 ± 6
AST	0-32 U/L	17 ± 4

Results expressed as mean ± standard deviation or n (%).

GTT: gestational transient thyrotoxicosis; fT3: free triiodothyronine; fT4: free thyroxine; TSH, thyroid-stimulating hormone; anti-TPO: anti-thyroid peroxidase antibody; anti-Tg: anti-thyroglobulin antibody; ALT: alanine aminotransferase; AST: aspartate transaminase.

The ECGs were obtained from 47 cases during thyrotoxicosis. In the overall analysis, 41 patients had sinus rhythm, four had sinus tachycardia, and two had sinus arrhythmia. None of the pregnant women had ventricular extrasystoles, ventricular hypertrophy, atrioventricular (AV) block, bundle branch block, ischemic changes, low QRS voltage, or loss of R progression. Detailed ECG data are presented in **Table 4**.

**Table 4:** Electrocardiogram findings in pregnant women with gestational thyrotoxicosis

Number of patients with ECG data	n = 47
Rhythm	Sinus tachycardia (n = 4)
	Sinus arrhythmia (n = 2)
	Sinus rhythm (n = 41)
Heart rate (beats/minute)	84 ± 13
Supraventricular extrasystole	n = 1
Cardiac axis	Normal (n = 42)
	Left axis (n = 4)
	Right axis (n = 1)
Atrial growth	One patient with right atrial enlargement
P wave	One patient with p pulmonale
PR interval (msec)	133 ± 14
Fascicular block	Left anterior fascicular block (n = 4)
	Left posterior fascicular block (n = 1)
QT interval (msec)	352 ± 27
QTc (msec)	413 ± 20
QRS duration (msec)	77 ± 8

Results expressed as mean ± standard deviation when not mentioned another way of presentation.

ECG: electrocardiogram; QTc: corrected QT interval.

The mean thyroid volume was 11.7 ± 5 mL, based on thyroid ultrasonography results performed at the time of presentation with thyrotoxicosis. Thyroid nodules were detected in 23 (46%) of the cases. The largest nodule detected was an isoechoic TI-RADS 3 nodule measuring 15×25×27 mm.

## DISCUSSION

This retrospective study, including 50 pregnant women with GTT, showed that serious obstetric complications, such as eclampsia/preeclampsia, GDM, preterm labor, and abortion, were not above the expected frequencies. In the detailed examination of ECGs, which were not evaluated in previous studies, we found an absence of serious, potentially life-threatening arrhythmias, although tachycardia was identified among the pregnant women. It is notable that the symptomatology and laboratory findings varied from one pregnant woman to another, but no serious adverse outcomes were observed.

Importantly, GTT remains the second most common cause of thyrotoxicosis during pregnancy. It is usually asymptomatic and resolves spontaneously. Although the differential diagnosis between GTT and Graves’ disease is often straightforward, it may be challenging in the absence of TRAb and in atypical GTT cases in which the suppression of TSH lasts longer than expected. In such cases, the differential diagnosis should be done carefully without delaying the diagnosis, thereby avoiding the possibility of unfavorable obstetric and perinatal outcomes related to Graves’ disease. In the present study, the TSH levels in 80% of the pregnant women reached the normal trimester-specific reference range for pregnancy at a mean of 18.3 ± 3.7 weeks. The latest recovery occurred in week 26 in a patient who was considered to have prolonged thyrotoxicosis due to increased hCG sensitivity of a mutant thyrotropin receptor, as described by Coulon et al. (^12^). However, this diagnosis cannot be established without a genetic analysis of the TSH receptor, and such analysis could not be performed in this case.

Inadequate control of thyrotoxicosis in pregnant women is associated with various risks, including spontaneous abortion, congestive heart failure, thyroid storm, preeclampsia, preterm delivery, low birth weight, and stillbirth (^13^). However, there are limited data on the adverse effects of GTT on pregnancy outcomes, and, generally, researchers have not reported adverse effects in this regard (^1^). However, there are also publications that have described adverse outcomes. In a retrospective study in which 208 pregnant women with GTT were compared with 6,317 healthy pregnant women, the gestational age at delivery was significantly lower in the GTT group (^1^). In another study conducted by Gürkan et al. in Türkiye, it was reported that GTT did not influence gestational age at delivery (^14^). In our study, the gestational age at delivery was 38 ± 1.8 weeks, and the frequency of preterm labor was 10%. However, three of the five pregnant women with preterm labor had multiple pregnancies. Based on the 10% frequency of preterm delivery reported in a large-scale study, we believe that the frequency of preterm delivery was not elevated in our group of pregnant women with GTT (^15^).

When the adverse effects on pregnancy were evaluated, our data showed no cases with adverse outcomes, such as eclampsia/preeclampsia or stillbirth. The frequency of abortions in this study was 4%. Considering the literature describing a miscarriage risk of approximately 15%, it can be interpreted that the frequency of miscarriage does not increase in pregnant women with GTT (^16^). In addition, the pregnant women with abortions in our study had reported a history of abortion in their previous pregnancies, suggesting other underlying causes.

Thyrotoxicosis has important effects on the heart, potentially causing tachycardia, atrial fibrillation, and heart failure (^17^). In a patient with thyrotoxicosis, the first findings examined on ECG data are rhythm and heart rate. Heart rate increases progressively during pregnancy, starting in the 4th week and reaching its highest level in the third trimester (^18^). In a multicenter study evaluating 1,041 pregnant women, the median heart rate at week 12 was 82 beats/minute (^19^). It has been reported that the heart rate does not exceed 115/minute, regardless of gestational age, and patients with a heart rate exceeding this threshold require further evaluation (^19^). The present study found only one pregnant woman with a heart rate of ≥ 115 beats/minute. This patient delivered a healthy baby at 38 weeks with no adverse maternal or neonatal outcomes.

Notably, GTT has been suggested to increase the frequency of GDM. A study conducted in our country reported that the frequency of GDM was higher in pregnant women with GTT compared with those without GTT (13.4% versus 4%, respectively) (^10^). In our study, the prevalence of GDM among the pregnant women who underwent oral glucose tolerance testing was 12.5% (n = 24). A comprehensive study on this topic, the TURGEP study, reported a GDM prevalence of 16% in Türkiye (^20^). Therefore, it can be interpreted that the prevalence of GDM did not increase in our group of patients with GTT. Of note, 12 pregnant women did not want to undergo oral glucose tolerance testing, and data were unavailable for 14 women. This indicates that at least one in three pregnant women either did not undergo (or refused) testing for GDM, a striking and concerning finding for our country, which has been defined as having a population at medium risk for GDM.

During pregnancy, hCG is released from the placenta. As hCG has been suggested to have thyrotropic effects, it may trigger thyroid nodule formation and growth during pregnancy (^21^). It has been reported that hCG levels are higher in pregnant women with GTT (^6^). Based on these data, an increase in the number or size of thyroid nodules may be expected in pregnant women with GTT. The nodule detection rate in pregnant women in our study was 48%, which is higher than the 29% prevalence reported in the literature (^22^). Although thyroid nodules were found in half of the pregnant women, none had nodules defined in the EU-TIRADS 5 category. Notably, fine-needle aspiration biopsy was not performed during pregnancy.

One of the strengths of the present study was the fact that a cardiologist evaluated the ECG results of almost all pregnant women. To our knowledge, there are no other publications in the literature in which ECGs of pregnant women with GTT were evaluated while they were thyrotoxic. Another important aspect of the present study was that almost all pregnant women underwent thyroid ultrasonography performed by an endocrinologist during thyrotoxicosis. The relevant limitations of this study include the inability to evaluate clearly the relationship between GTT and hyperemesis gravidarum due to its retrospective design. The criteria for > 5% weight loss and ketonuria used to diagnose hyperemesis gravidarum could not be used due to the unavailability of data in this respect. Although the frequency of hyperemesis gravidarum could not be determined in the present study, 62% of our patients with GTT reported nausea and vomiting intense enough to use antiemetic treatment.

In conclusion, this study reports unique detailed data regarding ECG and thyroid ultrasonography findings performed during thyrotoxicosis among pregnant women with GTT, which are important advantages relative to other studies in this field. Although some studies in the literature have associated GTT with adverse pregnancy outcomes, our data shows the absence of serious adverse effects (maternal or neonatal) in our group of pregnant women with GTT. However, larger prospective controlled studies with more extensive patient groups are necessary to confirm these results and provide greater clarity on the subject.

## Data Availability

the data supporting this study’s findings are available from the corresponding author upon reasonable request.
